# How to Treat Skin Quality: A Consensus‐Based Treatment Algorithm and Expert Guidance

**DOI:** 10.1111/jocd.70359

**Published:** 2025-08-23

**Authors:** Martina Kerscher, Kate Goldie, Cyro Hirano, Stephen Lowe, Kavita Mariwalla, Milton Moore, Je‐Young Park, Dusan Sajic, Sonja Sattler, Julieta Spada, Vasanop Vachiramon, Bianca Viscomi

**Affiliations:** ^1^ Division of Cosmetic Sciences University of Hamburg Hamburg Germany; ^2^ Goldie Aesthetics and Wellness London UK; ^3^ CD Clínica Dermatológica Rio de Janeiro Brazil; ^4^ MUSE Clinic Sydney New South Wales Australia; ^5^ Mariwalla Dermatology Islip New York USA; ^6^ Liv Dermatology and Aesthetics The University of the Incarnate Word Medical School San Antonio Texas USA; ^7^ Apgujeong Oracle Dermatology Clinic Seoul Korea; ^8^ Derma Skin Institute Guelph Ontario Canada; ^9^ Clinical Medicine McMaster University Hamilton Ontario Canada; ^10^ Rosenpark Klinik GmbH Darmstadt Germany; ^11^ Spada Dermatología y Estética Buenos Aires Argentina; ^12^ Division of Dermatology, Department of Medicine, Faculty of Medicine Ramathibodi Hospital Mahidol University Bangkok Thailand; ^13^ Bianca Viscomi Clínica Dermatológica São Paulo Brazil

**Keywords:** emergent perceptual categories, skin firmness, skin glow, skin quality, skin surface evenness, skin tone evenness, treatment algorithm

## Abstract

**Introduction:**

Skin quality can be described using four emergent perceptual categories (EPCs): skin tone evenness, skin surface evenness, skin firmness, and skin glow. While the publication in which the EPCs were originally described by Goldie et al. notes possible treatments for each EPC, there remains a need for a resource to guide clinicians in treatment selection when addressing EPCs in clinical practice.

**Methods:**

Twelve expert aesthetic physicians from across the globe participated in this EPC working group. A modified Delphi method was used to develop a treatment algorithm. First, panelists ranked a range of aesthetic treatments based on their ability to directly improve a given EPC. A draft algorithm was developed and evaluated during a moderated discussion. The treatment algorithm was then updated and reviewed by each individual participant, with the appropriateness of treatments listed for each EPC ranked using the RAND/UCLA scale. The algorithm was again updated based on this feedback and presented to the group for final review and approval during another virtual meeting.

**Results:**

The treatment algorithm developed by the working group is presented here alongside clinical pearls and preferred approaches for managing each EPC. It is the hope of the working group that the algorithm can be applied in real‐world clinical practice to improve patient skin quality and aesthetic outcomes.

**Conclusion:**

The presented guidance can serve as an approach to improving skin quality utilizing the framework of EPCs. The treatment algorithm may be applied to a range of skin types in practices across the globe.

## 1 Introduction

Skin quality is a key issue affecting patient self‐perception and is often a primary reason that patients vocalize for seeking aesthetic care. Thus, skin quality is also often a central component of positive treatment outcomes. Importantly, patients are increasingly aware of the need for expertise for improving their skin quality [1]. On a broader level, skin quality can be defined as skin that is healthy, undamaged, and youthful in appearance; however, a more granular approach is needed to develop patient‐specific treatment plans and to support inclusion of skin quality outcomes in clinical studies.

Goldie et al. described four emergent perceptual categories (EPCs) that can be used to describe skin quality across all ethnicities, age groups, and genders: skin tone evenness, skin surface evenness, skin firmness, and skin glow [2]. Importantly, the *perception* of good skin quality arises from these EPCs, but the source of the outward appearance of each individual EPC may arise from both the skin itself *and* the health and function of tissues beneath the skin. The original consensus paper provided some discussion around individual treatments that can address each EPC; however, an unmet need remains for a practical framework that can be used when approaching skin quality in real‐world clinical practice. Through an interactive process, the authors developed a treatment algorithm that can be applied to a range of skin types in practices across the globe by allowing customization and tailoring of treatments to both individual practices and individual patient needs. Here, the authors present the treatment algorithm for managing each EPC, alongside clinical pearls and expert advice for implementation.

## 2 Methods

The working group was chaired by Dr. Martina Kerscher and included 12 dermatologists and aesthetic physicians from nine countries: Argentina, Australia, Brazil, Canada, Germany, Republic of Korea, Thailand, United Kingdom, and United States. A modified Delphi method was used to develop and refine a treatment algorithm to manage EPCs [2, 3], with an objective to develop guidance for a universal approach to improve skin quality that could be applied to a range of skin types globally. By including a wide range of technologies, it is the authors' hope that the presented algorithm will facilitate implementation of evidence‐based care supported by clinical expertise to achieve the best outcomes for patients.

Using an asynchronous online platform, participants ranked aesthetic treatments based on the ability to directly address each of the four EPCs (skin tone evenness, skin surface evenness, skin firmness and skin glow). Rankings were collected in a blinded fashion, and a draft algorithm was developed based on these data. Next, the working group met virtually for a moderated 2.5‐h meeting in which these data were reviewed along with the draft algorithm, and feedback and clarification were provided. The treatment algorithm was updated based on this meeting, and a revised version was presented for review using an online asynchronous platform. Members were asked to review the algorithm and provide commentary on its content, rating the appropriateness of the listed treatments and approaches for each EPC using the RAND/UCLA scale (1 being inappropriate and 9 being highly appropriate) [4]. Responses were blinded. Median appropriateness scores for individual treatments for specific EPCs were calculated, and only treatments with a mean score of > 6.5 were included in the final algorithm. If the median was > 6.5, but more than two panelists provided a score below this threshold, additional feedback was solicited, and the treatment was either removed or a qualifying statement added to the discussion. To ensure broad applicability, appropriateness was ranked for the algorithm itself, including the ability to address skin quality EPCs, key concerns for patients within varying geographic regions, and practicality for implementation in a clinical setting. Advisors provided qualitative feedback to assist in algorithm revision. The algorithm was again updated after this process and presented to the group for final review and approval as part of a 2‐h virtual board. This advisory board did not include human research subjects, and Institutional Review Board Approval was not required.

## 3 Results

**FIGURE 1:**
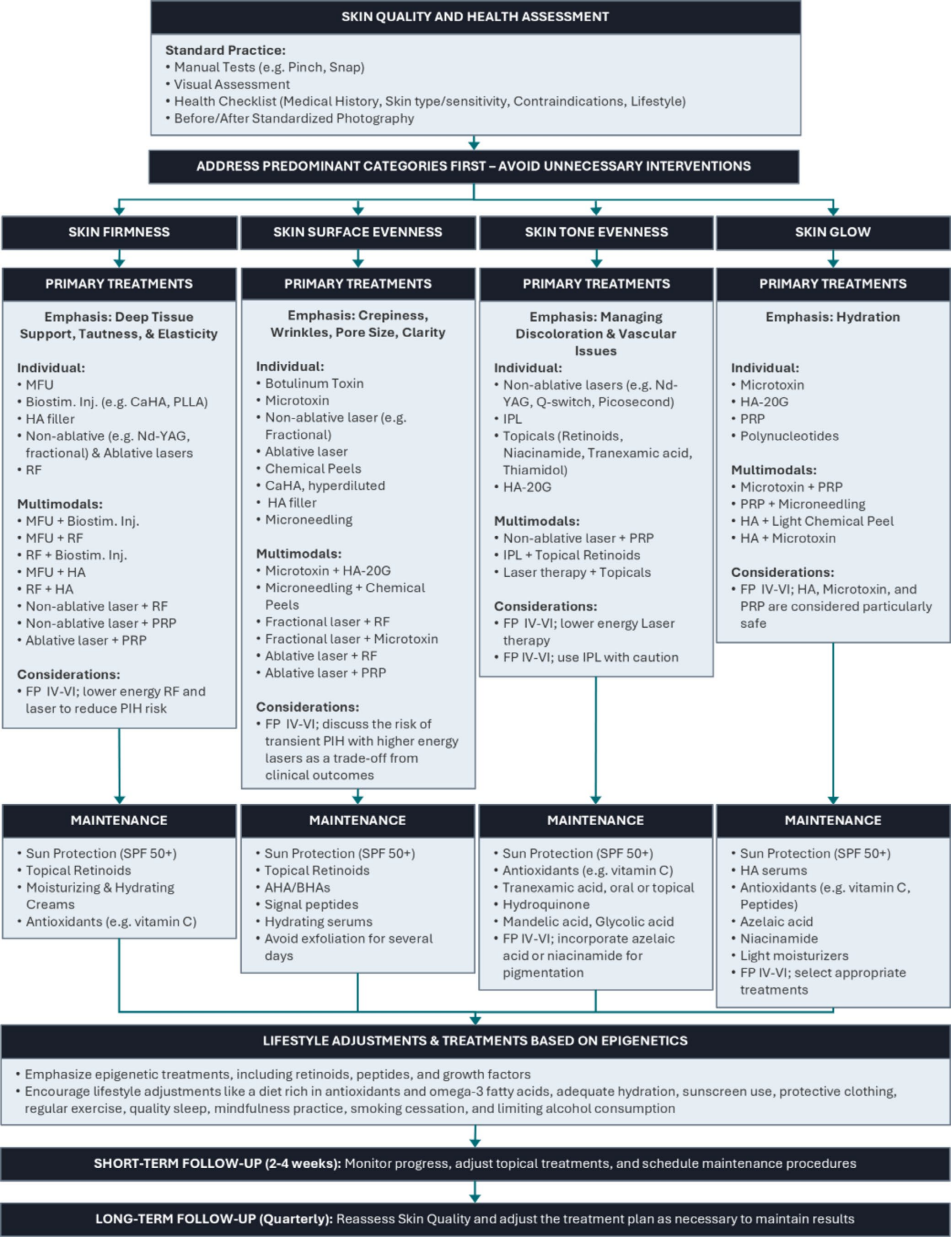
Algorithm for management of EPCs.

The working group developed a treatment algorithm containing individual and combination treatments that can address individual EPCs (Figure 1). As part of the development process, several important topics surfaced for interpretation and implementation. Overall guiding principles for implementation, the foundational nature of the firmness EPC, and practical guidance on patient evaluation were all identified as critical. In addition, a more in‐depth discussion of the top treatments for each EPC, as well as the emergence of combination treatment as a gold standard for clinical care is detailed below.

### 3.1 Algorithm Structure

The algorithm begins with a health assessment. The skin is an indicator of overall health [5]; thus, prior to managing the EPCs, it is best to manage existing inflammatory conditions and address any underlying health issues which could affect the skin's appearance. In addition to manual tests and visual assessments, a detailed patient history of skin sensitivity and healing dynamics should be collected. Lifestyle factors that could impact the skin should be addressed (e.g., smoking, hydration, sleep, sunscreen). Panelists wish to emphasize the importance of diligent, standardized clinical photography, which is critical for patients to be able to appreciate change.

Overall, the success of this algorithm depends on individual clinician experience, careful patient assessment, and customization based on individual patient needs, lifestyle and habits, decade of life, and/or phototype. In the presented algorithm, treatments are categorized based on the EPC they most *directly* affect; however, treatments listed for a given EPC can have overlapping effects on other EPCs (e.g., a treatment to improve firmness may also impact skin surface evenness and skin tone). Because comparative data between suggested treatments are not generally available, the order in which they are listed is a reflection of participant ranking of appropriateness, which is based on clinical experience.

The algorithm includes preferred treatment combinations with important procedural information to reduce safety risks in patients with skin of color. There was strong consensus that combination treatment is emerging as the gold standard and that treatments should be intentionally combined based on mechanism of action, the tissue layer where activity predominates, and evidence base. Treatments may need to be modified for sensitive skin types or patients with darker phototypes. To optimize safety, pre‐ and post‐procedure treatments/care must be tailored to individual patient needs and device settings appropriately modified. There was strong consensus that ongoing maintenance can increase the quality and duration of results and that the impact of lifestyle modifications should not be underestimated. When planning treatments, it is important to take into account patient comfort and experience, tolerance for pain during procedures, and required downtime [6]. Procedure cost is a significant aspect of treatment selection that is not covered here but is of central importance for patients.

### 3.2 Guiding Principles for Implementation

In addition to sound clinical judgment throughout the treatment course, general guiding principles for applying the treatments listed for each EPC include the following:

#### 3.2.1 Pretreat the Skin

- It is critical to treat existing skin conditions (e.g., acne, eczema, dermatosis papulose nigra/seborrheic keratosis) or refer for medical issues that could impact skin quality prior to using devices to improve aesthetics. When inquiring about skin sensitivity, clinicians should ask about recent exposures or environmental causes, as well as possible causes of sensitive skin, including those related to the gut, specific foods, dairy intolerance, gluten intolerance, or celiac disease. In some cases, probiotics and diet can have a profound effect on skin health and healing capacity, which should be optimized prior to treatment.

#### 3.2.2 Reinforce

- Repair the skin barrier with topicals before any procedure, if needed. This can include selection of an appropriate cleanser and application of products like topical HA, ceramides, Vitamin B such as niacinamide and D‐panthenol; however, topicals should be selected based on individual patient needs, including phototype and decade of life. There was strong consensus that both pre‐nd post‐treatment care can maximize results and aesthetic practitioners can help patients navigate topicals, a crowded landscape where product marketing can be misleading and advantages of particular ingredients overstated. For example, in order for topical peptides to be effective, they must be the right size, molecular weight, and charge in order to make it into the dermis as well as be stable within the formulation [7]. In addition, low molecular HA applied topically can actually be pro‐inflammatory, rather than anti‐inflammatory and regenerative [7, 8]. The skin should also be reinforced using topicals following procedures, as part of a customized maintenance program.

#### 3.2.3 Go Slow

- There was strong consensus that tolerance to treatments should be built over time to avoid overchallenging the skin and unnecessary side effects. Especially in patients with sensitive skin or darker Fitzpatrick Skin Types (FST), treatments should be spaced out over time and both the density and depth of energy‐based devices (EBDs) and microneedling should be carefully considered. Lasers and EBDs have distinct mechanisms of action, which make them better suited to different skin types and aesthetic concerns [9].

#### 3.2.4 Be Proactive in Preventing and Treating Post‐Inflammatory Hyperpigmentation

- In all patients, especially those with sensitive or more pigmented skin (FST IV‐VI), post‐inflammatory hyperpigmentation (PIH) must be diligently prevented and managed. Avoid PIH through customization and caution with EBDs in higher phototypes. Appropriate/conservative chemical peels, exfoliation, and injectables remain very safe in patients with skin of color. Post‐procedure interventions should focus on recovering the skin barrier using topical gels or creams designed for that purpose or additional interventions such as LED application or platelet‐rich plasma (PRP) to expedite healing and ensure adequate skin hydration. For all patients, but particularly for more sensitive skin types, post‐procedure care should include diligent avoidance of sun and use of sunscreen. Consider potent topical steroids or low‐dose systemic steroids for a short period in very high‐risk patients. Panelists noted that the highest risk procedures are intense pulsed light (IPL), lasers, radiofrequency (including fractional and microneedling), and chemical peels. For skin of color, it is important to be educated and experienced before treating and to refer patients if needed. In order to repair conditions like pigmented acne scars, one must use impactful settings for impactful results, albeit with reduced density. Prior to procedures, oral or topical tranexamic acid, hydroquinone, isobutylamido thiazolyl resorcinol, or azelaic acid may be used, depending on skin type, and post‐procedure care should include aggressive skin barrier repair until full recovery along with corticosteroids twice a day for no more than 1 week, depending on the skin type.

### 3.3 Firmness Is a Foundational EPC

While the EPCs describe skin quality, they more specifically describe the *emergent* properties of skin quality, which can be affected by age‐related changes in deeper layers. Thus, interventions aimed at tissue layers beneath the skin can impact the skin's outward appearance. For example, tissue laxity along the jawline can be addressed via direct management of the skin using energy‐based devices; however, treatments such as filler injection along the mandible or injection of the platysma with botulinumtoxinA (BoNT‐A) can also affect the outward appearance of skin quality, making it seem less lax, firmer, and more elastic. These additional interventions do not convey these properties to the skin itself, but they do give the appearance of these qualities (i.e., the emergent property). Even the most aggressive and direct management of skin quality will have an incomplete effect on a given EPC if other tissue layers are not addressed.

**FIGURE 2:**
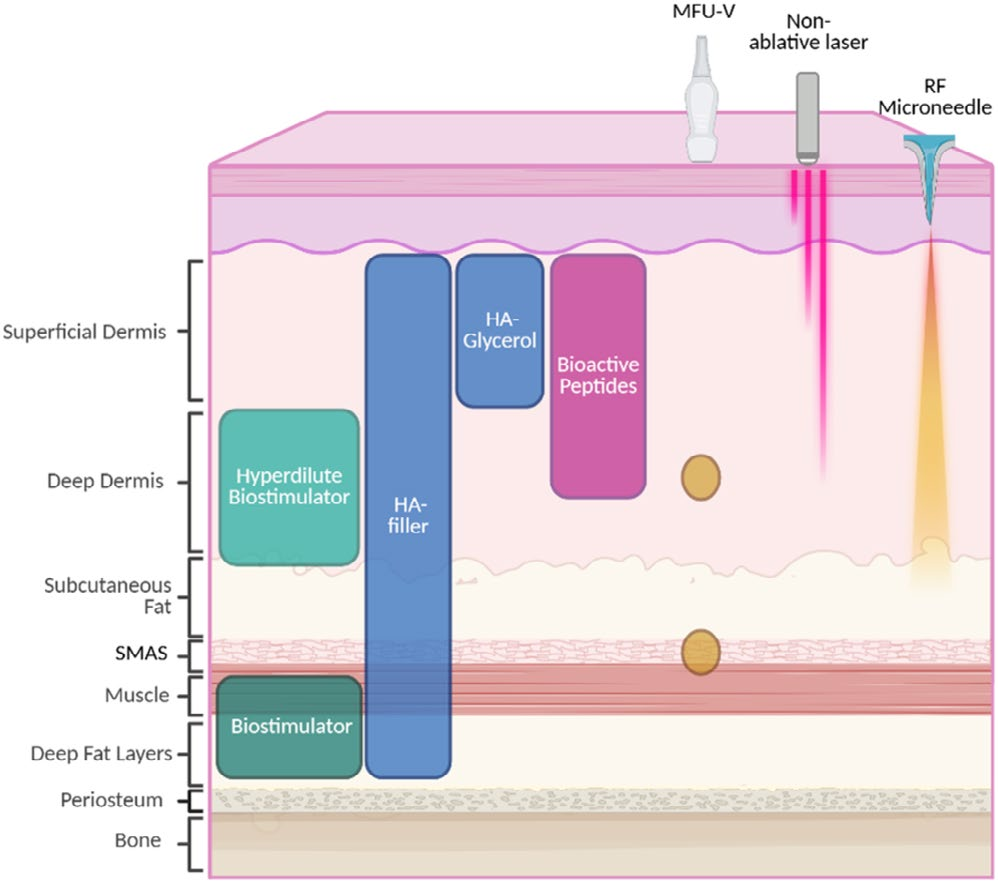
Management of firmness can include treatments in multiple tissue layers.

There was a strong consensus that skin firmness, which includes elasticity, tautness, and hydration, is a foundational EPC and that its management is requisite for effective management of the other 3 EPCs. Without firmness, management of EPCs like skin surface glow will have a limited effect on global appearance, which is most often the outcome sought by patients. In order to manage firmness, each layer of deeper tissue support should be addressed. This need is the basis for inclusion of fillers in the algorithm as treatments that improve firmness. In Figure 2 approaches to improving firmness are shown. Firmness should be optimized in order to improve stability and homeostasis—if the skin is too tight or too rigid, the health of cells within the skin declines.

### 3.4 Patient Assessment

In the original manuscript in which the EPCs were described by Goldie and colleagues, detailed descriptions of clinical and research tools for measuring aspects of each EPC were provided [2]. In clinical practice, however, many of these tools are not as commonly used. In Table 1 an abbreviated guide is provided for real‐world clinical assessment of each EPC.

**TABLE 1:** Initial patient assessment.

EPC	Skin firmness	Skin surface evenness	Skin tone evenness	Skin glow
Key emergent properties	Elasticity, tautness, and hydration	Pores, wrinkles, crepiness, and clarity	Uniformity in pigmentation without erythema	Also understood as radiance, vibrancy
Notes	Assess and manage underlying conditions first Aggressively manage preexisting inflammation Pre‐treatment care can affect overall outcome			
	Foundational EPC, ideally managed first	Can be impacted by improving skin firmness, but additional procedures based on the predominant surface issue may be needed	Manage vascular and pigmentary issues separately	Most difficult to quantify Managed after the other 3 EPCs are optimally addressed Affected by lifestyle changes
Clinical evaluation checklist	Skin Laxity Visible dehydration Contribution of lack of underlying support (loss of bone, fat) Snap and Pinch test [10] for elasticity and tautness Hormone status	Pore size and number Skin sebum production Crepiness Dynamic lines Static lines Deeper folds and wrinkles Scars Hair Skin clarity Stretch marks	Vascular unevenness Pigment unevenness Check depth of pigment and skin thickness Sun exposure Rosacea or ongoing inflammatory issues	Hydration Optimized texture Review home care routine
Treatment selection	Treatment combinations can have overlapping effects and improve multiple EPCs Treatments should be selected based on patient priorities and highest impact on global improvement Complementary mechanisms of action can be selected to improve outcomes and have broadest effect Post‐treatment maintenance and at‐home care can improve the nature and longevity of results			

### 3.5 Recommended Treatments

The recommended treatments for each EPC within the algorithm are ordered based on appropriateness rankings and later discussion by the working group. Because there are very little comparative data for these treatments, rankings are based primarily on panelist experience and preference in their clinical practice. All treatments must be tailored to individual patient needs. Appropriateness rankings for individual treatments are shown in Tables S1 and S2, and appropriateness rankings for the algorithm are shown in Table S3.

#### 3.5.1 Skin Firmness

There was strong consensus that microfocused ultrasound with visualization (MFU‐V, Ultherapy, Ulthera Inc., Raleigh, NC, a company of the Merz Aesthetics group), dilute and hyperdilute calcium hydroxylapatite‐carboxymethylcellulose (CaHA, Radiesse, Merz North America Inc., Franksville, WI), and ablative fractional lasers are the preferred treatments to address skin firmness. MFU‐V directly addresses elasticity and tautness through improving laxity and is safe for all skin types because it does not cause hyperpigmentation [11, 12]. While MFU‐V was ranked as the most appropriate for improving firmness, it also improves surface evenness through improvements in skin crepiness, as the skin envelope is strengthened and tissue remodeled, with increased collagen and elastin reducing skin laxity [12, 13, 14, 15, 16]. Effects on tone for MFU‐V have also been noted due to reduced skin roughness and improvements in evenness, texture, smoothness, and pigmentation [17]. Skin roughness and pore size have been shown to be significantly reduced [17].

Both diluted and undiluted CaHA stimulate neocollagenesis, elastogenesis, proteoglycans, and neovascularization, leading to improved overlying skin quality [18, 19, 20, 21]. CaHA microspheres stimulate fibroblasts in a contact‐dependent manner, and dilute and hyperdilute CaHA can be delivered as a thin layer in the deep subdermal layer to improve overlying skin quality without providing volume [22, 23]. Hyperdilute CaHA increases skin thickness, elasticity, and pliability, which are important elements of firmness [19]. Treatments can be administered in the face and body and are most effective when administered as a series of two to three treatments spaced at least 6 weeks apart [24].

Fractional lasers can be used to improve firmness and elasticity [25]. While radiofrequency and IPL can provide improvement, the magnitude of improvement with ablative lasers appears to be greatest [25]. Fractional laser treatment generates bulk heating of the skin, which results in thermal coagulation, induction of the wound‐healing response, and formation of collagen and elastin [26]. This can rejuvenate the skin and has been reported to prevent formation of non‐melanoma skin cancers, thereby addressing not just skin firmness but also skin health [27]. Non‐ablative lasers and those that target water rather than melanin are safest for those with skin of color, and technologies that spare the epidermis may be best for patients with darker skin phototypes.

There was a strong consensus that combination approaches to firmness are preferred. Treatments should be combined based on complementary mechanisms (treating multiple tissue layers), clinical compatibility, patient needs, and availability. MFU‐V with biostimulatory injectables (hyperdilute) was frequently cited as a preferred treatment, and there is evidence that this combination has an additive effect in the face and body [28, 29, 30]. Some evidence suggests that administering MFU‐V first followed by hyperdilute CaHA 6 weeks later is associated with the best outcomes [31], but it is also common to deliver CaHA first to induce fibroblast proliferation followed by MFU‐V 6 weeks later.

When radiofrequency devices and MFU‐V are used together, they can treat tissue at different depths, further improving results. Similar to MFU‐V, application of radiofrequency with biostimulatory injectables can bolster results [32, 33]. MFU‐V and radiofrequency can also be combined with HA fillers or undiluted biostimulatory fillers; however, here the purpose of the filler would be to provide firmness in deeper layers via the lifting capacity of the filler, while using the EBD to strengthen the skin envelope prior to filler injection to improve the efficiency of the filler and improve firmness through multiple levels of treatment [34, 35].

#### 3.5.2 Skin Surface Evenness

Preferred treatments to address skin surface evenness include fractional lasers, RF‐microneedling, dilute/hyperdilute biostimulatory fillers, microtoxin injections, and Cohesive Polydensified Matrix hyaluronic acid (CPM‐HA 20G, Belotero, Anteis S.A., Plan‐les‐Ouates, Switzerland, a company of the Merz Aesthetics group) [35]. Fractional lasers, while also an important tool for improving firmness, can also reduce fine lines and improve texture, thereby improving surface evenness [25]. RF microneedling is also an important tool for improving skin texture through induction of skin and adipose tissue remodeling, and also through tightening the underlying septal network [36, 37]. Hyperdilute fillers are an established means for skin tightening, which can reduce skin laxity and resultant surface irregularities in the face and body [24, 38]. Additionally, the use of MFU‐V with dermal transducer (i.e., 1.5 mm) alone or in combination with low‐crosslinked CPM‐HA filler minimized enlarged facial pores [39]. Microtoxin injections, in which BoNT‐A is injected intradermally as multiple microdroplets, have a dual effect [40]. Microtoxin can act on muscles where they insert into the skin, addressing fine lines and/or creating lift through action on depressor muscles as well as on nonneuronal cholinergic pathways [41]. Through this action, microtoxin can reduce sebum production and pore size and has also been used to improve the appearance of scars [40, 42, 43]. Because the dermis contains a higher number of dendritic cells than the muscle, there is an increased likelihood of antigen capture and subsequent immunologic response [44]. For this reason, panelists prefer using incobotulinumtoxinA (INCO, Xeomin, Merz Pharmaceuticals GmbH, Frankfurt, Germany) for this application [45]. Administration of CPM‐HA20G, a combination of high molecular weight HA (20 mg/mL) and glycerol (17.5 mg/mL) recovers skin hydration, elasticity, and firmness, which can improve skin surface evenness and address fine lines as well as reduce pore size [46, 47, 48, 49]. Further, CPM‐HA20G is a potential hydrating pretreatment to prepare the skin for additional interventions.

While botulinum toxins are not specifically noted to address skin surface evenness, there was strong consensus that management of dynamic lines with BoNT‐A is an important part of any holistic approach to rejuvenation and management of skin surface evenness [50]. As the number of uses for BoNT‐A continues to expand to manage the aesthetics of the upper and lower face, as well as the neck, doses used will be higher than “aesthetic doses” associated with a low incidence of neutralizing antibodies of between 0% and 1% [51, 52]. For this reason, INCO is a preferred choice for panelists because it lacks hemagglutinin proteins, which may serve as an adjuvant, increasing the risk of immunogenicity [51, 52]. Across all recommended combination treatments, modalities are paired to act on the deeper layers of the skin as well as the superficial dermis and are paired based on complementary mechanisms of action. For example, fractional RF and microneedling act on different layers of the skin and through different mechanisms, thereby activating wound healing through different means and improving overall outcome [53].

While it is important to educate patients with darker phototypes about the risk of hyperpigmentation associated with lasers, this discussion is not necessarily about avoidance altogether but rather managing risk and tradeoffs for the best clinical outcomes. Panelists with experience treating patients with darker skin types noted that impactful results require impactful settings, and that it is important to understand the limits of the device being used. Clinicians should also be aware of their own limitations.

#### 3.5.3 Skin Tone Evenness

When addressing skin tone evenness, it is important to first assess the contribution of pigment vs. the contribution of vascular issues or erythema. Treatment selection should be based on the primary issue to be addressed, and devices with overlapping effects should be selected when needed. To address pigment, advisors recommend laser treatment (Q‐switched, picosecond), IPL, or superficial chemical peels [54, 55]. If pigment is from photodamage can add ablative lasers like CO_2_ or erbium can be used. Most often, in practice, peels are used to prepare the skin for additional treatment with devices or mesotherapy. For vascular issues, advisors most prefer laser therapy, IPL, microtoxin, and CPM‐HA20G. For microtoxin, there is evidence that rosacea, as well as other skin tone issues can be improved [36, 56]. For CPM‐HA20G, the primary function of the HA is to attract water and improve hydration, which has a positive effect on erythema.

When discussing multimodal treatment, panelists agree that topical treatments can be used in combination with energy‐based devices to achieve optimal results. While platelet‐rich plasma (PRP) is listed for combination use, it is utilized less to improve the efficacy of treatment and more to accelerate healing [57].

While IPL is effective for both pigmentary and vascular issues, advisors agree that they see far more complications from other practices with IPL than from any other laser, highlighting the importance of the treaters experience with this device. As with treating skin surface evenness, it is important to be aware of the need for modified treatment settings (i.e., energy and pulse duration) for skin of color.

#### 3.5.4 Skin Glow

There was strong consensus among the authors that the other EPCs should be addressed prior to skin glow. Without skin firmness, surface evenness, and skin tone evenness, improved glow will have a limited impact on overall appearance. Though skin glow is difficult to explain to patients and capture in before and after photographs, radiant skin is important for facial attractiveness [58]. Importantly, many of the key treatments for glow can be easily incorporated into combination treatment approaches to improve results, including CPM‐HA20G, INCO (either administered as microtoxin or through secondary effects following intramuscular injection), and polynucleotides [59]. Each of these treatments improves glow through a slightly different mechanism. CPM‐HA20G improves radiance via hydration and through the actions of glycerol; microtoxin improves glow via non‐cholinergic acetylcholine (ACh) pathways and improvement of light reflection through sebum reduction and improvement of the skin's surface; and polynucleotides improve glow through hydration with secondary effects on stimulation of collagen, elastin, and growth factors, all of which improve skin resilience [43, 59, 60]. For patients with darker phototypes, HA in combination with PRP is considered by panelists to be particularly safe. For all patients, when using IPL and retinol, retinol should be stopped a week before IPL and not resumed until a week after treatment.

### 3.6 Multimodal Treatment Is the Gold Standard

There was a strong consensus that combination treatment is becoming the standard of care [34, 61]. Combinations can be selected based on complementary mechanisms of action and activity in different tissue layers and should be selected so that secondary effects permit management of multiple EPCs simultaneously. The authors acknowledge the need for data comparing combination approaches. Currently, it is difficult to define which combinations are more effective than others; however, there is evidence that the key recommended combinations like MFU‐V or radiofrequency with biostimulatory fillers are effective [62, 63, 64] As the landscape of aesthetic medicine has shifted to one focused on proactive and consistent management with non‐surgical treatments, combination treatments, especially when maintained over time with adequate home care, topicals, and in‐office maintenance treatment, can shift the outward trajectory of aging.

## 4 Discussion

Utilizing a modified Delphi method, the working group developed a treatment algorithm for the management of EPCs in clinical practice. Treatments were included based on appropriateness rankings and group consensus, with preferred treatments listed first. Though the treatments for each EPC are shown in separate columns and are categorized based on the EPC they most directly impact, effects overlap and a given treatment or combination can address multiple EPCs. There was strong consensus that combination treatments, particularly those that span multiple EPCs, are emerging as the gold standard. Treatment planning should consider additive and synergistic effects and/or impacts across multiple tissue layers, and multimodal treatment should be considered when possible. When discussing treatment with patients, the EPCs can be used as a guide to improve understanding of outcomes.

While the treatment algorithm can be used as a general guide, good clinical judgment should be exercised, and both decade of life and phototype should influence treatment selection. Beyond treatment selection itself, treater awareness of risk factors as well as familiarity with the device being used, in particular how to adjust treatment settings, is critical for success. In particular, when managing patients with sensitive skin or patients with skin of color, the treatment approach should take into account individual skin biology. The participants emphasize the need for referral if the treating clinician is not comfortable managing a patient with PIH or other inflammatory risks.

The participants noted that while nonsurgical approaches are often discussed as surgical alternatives, in reality these two approaches complement one another. Minimally invasive procedures cannot substitute for surgery when it is needed, but minimally invasive procedures can achieve outcomes that are distinct from but complementary to those that can be achieved with surgery. For example, a surgical result can be improved by replenishing facial firmness and support with fillers, but neither one of the interventions can substitute for the other.

When discussing topical treatments and the need for appropriate at‐home maintenance, advisors agreed that it is important to help patients navigate the complexities of topical treatments. There are many ingredients touted as rejuvenating, only some of which are validated, and some can be counterproductive for improving skin health. Aesthetic clinicians can help to direct patients to clinically proven skin ingredients to support their goals.

A possible shortcoming of this algorithm is that it is based on clinician perceptions of efficacy only. It would be of interest to develop an understanding of patient perceptions of outcomes using combination treatments as well as aspects of patient experience (e.g., cost, discomfort associated with the procedure) that impact willingness to undergo initial or maintenance treatment. In addition, the working group elected not to include exosomes and growth factors within this algorithm. Though there is promise for reducing downtime and improving skin quality, to date there is insufficient data, both short‐ and long‐term, to place this treatment within the algorithm [65, 66]. The working group noted that while it can be tempting to focus on novel treatments and approaches, it is important not to forget that straightforward and easy approaches are available (e.g., subcision to address scars or chemical peels to address under‐eye circles) [67, 68]. Aesthetics is a rapidly changing field, and the modalities listed within the algorithm are based on currently available evidence and technology, and recommendations may change as novel treatments are developed.

## 5 Conclusions

The guidance presented here can serve to inform an approach to improving skin quality within the EPC framework. The treatment algorithm may be applied to a range of skin types in practices across the globe.

## Conflicts of Interest

Dr. Kerscher is an advisory board member, consultant, and clinical trial investigator for Allergan/AbbVie, Croma, Galderma, Ipsen Innovation, Merz Aesthetics, Neauvia/Matex, and Nordberg. Dr. Goldie and Dr. Park are consultants and speakers for Merz Aesthetics. Dr. Hirano is a speaker for Merz Aesthetics, L'Oréal, Neauvia, EVO Pharma, and LMG. Dr. Lowe is a consultant and speaker for Merz Aesthetics and Sciton laser. Dr. Mariwalla and Dr. Moore are consultants for Merz Aesthetics. Dr. Sajic received grants/research support from AbbVie, Bausch, Boehringer‐Ingelheim, Concert, Croma Pharma, Derma Research Group, Leo Pharma, Novartis, Janssen, Laboratoires Pierre Fabre, Merz, UCB, and Sun Pharma; served on the speakers bureau or received honoraria from AbbVie, Actelion, Arcutis Biotherapeutics, Alma Lasers, Bausch, Concert, Celltrion, Derma Research Group, Galderma, FillMed Pharma, InMode, Lilly, Laboratoires Pierre Fabre, Leo, L'Oréal, Novartis, Merz Aesthetics, Janssen, Pfizer, Sun Pharma, and UCB; and received consulting fees from AbbVie, Actelion, Arcutis Biotherapeutics, Alma Lasers, Bausch, Concert, Derma Research Group, FillMed Pharma, Galderma, InMode, Incyte Biosciences, Lilly, Laboratoires Pierre Fabre, Leo, Novartis, Pfizer, Janssen, and Sun Pharma. Dr. Sattler received research support from and conducted clinical trials for Merz Aesthetics, Allergan Aesthetics, Hallura, LC Chem, Croma, and Crown Laboratories; and acted as a KOL, speaker, advisor, or investigator for Merz Aesthetics, Allergan Aesthetics, ICA Navigation Systems, Crown Aesthetics, Hallura, and IBSA. Dr. Spada and Dr. Viscomi are Merz Aesthetics Global Speakers. Dr. Vachiramon is an investigator for AbbVie, Biersdorf, Fillmed, L'Oreal, Merz Aesthetics, Viam Global; and is a speaker for AbbVie, Biersdorf, Fillmed, Galderma, L'Oreal, Merz Aesthetics, and Viam Global.

## Supporting information


**Table S1.** Appropriateness rankings for skin firmness and skin surface evenness treatments included in the algorithm (treatments with a mean score of > 6.5). Note that following this round of ranking, the algorithm was modified further based on feedback from group discussion.
**Table S2.** Appropriateness rankings for skin tone evenness and skin glow treatments included in the algorithm (treatments with a mean score of > 6.5). Note that following this round of ranking, the algorithm was modified further based on feedback from group discussion.
**Table S3.** Appropriateness rankings for the presented algorithm.

## Data Availability

The data that supports the findings of this study are available in the Tables S1–S3 of this article.
