# Supplementary material for: How to Treat Skin Quality: A Consensus‐Based Treatment Algorithm and Expert Guidance
Source: J Cosmet Dermatol. 2025 Aug 23;24(Suppl 4):e70359. doi: 10.1111/jocd.70359 (PMC12374567; doi:10.1111/jocd.70359)
Supplement: Supplementary file 1 — Table S1. Appropriateness rankings for skin firmness and skin surface evenness treatments included in the algorithm (treatments with a mean score of > 6.5). Note that following this round of ranking, the algorithm was modified further based on feedback from group discussion. Table S2. Appropriateness rankings for skin tone evenness and skin glow treatments included in the algorithm (treatments with a mean score of > 6.5). Note that following this round of ranking, the algorithm was modified further based on feedback from group discussion. Table S3. Appropriateness rankings for the presented algorithm. [file JOCD-24-e70359-s001.docx]

**Supplementary Table 1.** Appropriateness rankings for skin firmness and skin surface evenness treatments included in the algorithm (treatments with a mean score of >6.5). Note that following this round of ranking, the algorithm was modified further based on feedback from group discussion.

|  |  |  | **PERCENTAGE** | | |
| --- | --- | --- | --- | --- | --- |
| **SKIN FIRMNESS** | **MEAN** | **MEDIAN** | **1-3** | **4-6** | **7-9** |
| ***TREATMENT*** |  |  |  |  |  |
| MFU + Biostimulatory Injectables | 9.0 | 9 | 0% | 0% | 100% |
| Microfocused Ultrasound (MFU) | 8.8 | 9 | 0% | 0% | 100% |
| Biostimulatory Injectables (e.g. CaHA, PLLA) | 8.7 | 9 | 0% | 8% | 92% |
| MFU + Radiofrequency (RF) | 8.5 | 9 | 0% | 0% | 100% |
| RF + Biostimulatory Injectables | 8.4 | 9 | 0% | 0% | 100% |
| MFU + HA | 7.8 | 8.5 | 0% | 25% | 75% |
| RF + HA | 7.3 | 7 | 0% | 17% | 83% |
| Hyaluronic acid (HA) filler | 7.1 | 7 | 0% | 33% | 67% |
| Non-ablative laser + RF | 6.6 | 6.5 | 0% | 50% | 50% |
| Non-ablative lasers (e.g. Nd-YAG, fractional) | 6.2 | 6.5 | 8% | 42% | 50% |
| Non-ablative laser + PRP | 5.5 | 5 | 8% | 67% | 25% |
| ***MAINTENANCE*** |  |  |  |  |  |
| Sun Protection (SPF 50+) | 8.9 | 9 | 0% | 0% | 100% |
| Topical Retinoids | 8.5 | 9 | 0% | 0% | 100% |
| Moisturizing/Hydrating creams | 7.7 | 8 | 8% | 0% | 92% |
| **SKIN SURFACE EVENNESS** |  |  |  |  |  |
| ***TREATMENT*** |  |  |  |  |  |
| Ablative lasers | 8.7 | 9 | 0% | 0% | 100% |
| Microneedling (radiofrequency (RF), regular) | 8.3 | 8.5 | 0% | 0% | 100% |
| Non-ablative lasers (e.g. Fractional) | 8.3 | 8.5 | 0% | 0% | 100% |
| Fractional laser + microtoxin | 8.3 | 9 | 0% | 17% | 83% |
| Ablative laser + PRP | 8.2 | 9 | 0% | 25% | 75% |
| Botulinum Toxin | 8.1 | 8.5 | 0% | 17% | 83% |
| Fractional laser + RF | 7.8 | 8 | 0% | 25% | 75% |
| Chemical Peels | 7.6 | 8 | 0% | 17% | 83% |
| Ablative laser + RF | 7.6 | 8 | 0% | 25% | 75% |
| Hyaluronic acid (HA) filler | 7.5 | 8.5 | 0% | 33% | 67% |
| CaHA (hyperdilute) | 7.4 | 8 | 0% | 17% | 83% |
| Microneedling + Chemical Peels | 7.3 | 8 | 8% | 8% | 83% |
| ***MAINTENANCE*** |  |  |  |  |  |
| Topical Retinoids | 8.8 | 9 | 0% | 0% | 100% |
| Sun Protection (SPF 50+) | 8.7 | 9 | 0% | 8% | 92% |
| Signal peptides | 8.1 | 9 | 0% | 25% | 75% |
| Hydrating Serums | 7.8 | 8.5 | 0% | 17% | 83% |
| Avoid exfoliation for several days | 6.6 | 7 | 17% | 33% | 50% |

**Supplementary Table 2.** Appropriateness rankings for skin tone evenness and skin glow treatments included in the algorithm (treatments with a mean score of >6.5). Note that following this round of ranking, the algorithm was modified further based on feedback from group discussion.

|  |  |  | **PERCENTAGE** | | |
| --- | --- | --- | --- | --- | --- |
| **SKIN TONE EVENNESS** | **MEAN** | **MEDIAN** | **1-3** | **4-6** | **7-9** |
| ***TREATMENT*** |  |  |  |  |  |
| Non-ablative lasers (Q-switch, Picosecond) | 9.0 | 9 | 0% | 0% | 100% |
| Intense Pulsed Light (IPL) | 8.7 | 9 | 0% | 8% | 92% |
| Topicals (Retinoids, Niacinamide, Tranexamic Acid, Thiamidol) | 8.0 | 8.5 | 0% | 17% | 83% |
| IPL + Topical Retinoids | 7.8 | 9 | 8% | 8% | 83% |
| Laser Therapy + Topicals | 7.7 | 9 | 8% | 8% | 83% |
| Non-ablative laser + Platelet-Rich Plasma (PRP) | 7.0 | 7 | 0% | 25% | 75% |
| Hyaluronic Acid (HA) Filler with glycerol | 5.8 | 6.5 | 17% | 33% | 50% |
| ***MAINTENANCE*** |  |  |  |  |  |
| Sun Protection (SPF 50+) | 9.0 | 9 | 0% | 0% | 100% |
| Antioxidants (e.g. vitamin C) | 8.3 | 9 | 0% | 8% | 92% |
| Tranexamic acid, oral or topical | 7.9 | 9 | 8% | 17% | 75% |
| FP IV-VI; incorporate azelaic acid or niacinamide for pigmentation control | 7.7 | 8.5 | 0% | 25% | 75% |
| **SKIN GLOW** |  |  |  |  |  |
| ***TREATMENT*** |  |  |  |  |  |
| Hyaluronic Acid (HA) Filler with glycerol | 8.7 | 9 | 0% | 8% | 92% |
| HA + microtoxin | 8.3 | 9 | 0% | 17% | 83% |
| HA + Light Chemical Peel | 8.0 | 9 | 0% | 25% | 75% |
| Botulinum Toxin (microtoxin) | 7.8 | 8 | 0% | 17% | 83% |
| Botulinum Toxin + PRP | 7.2 | 7.5 | 8% | 8% | 83% |
| PRP + Microneedling | 7.2 | 8 | 8% | 17% | 75% |
| Platelet-Rich Plasma (PRP) | 6.9 | 7 | 8% | 17% | 75% |
| ***MAINTENANCE*** |  |  |  |  |  |
| Sun Protection (SPF 50+) | 9.0 | 9 | 0% | 0% | 100% |
| Antioxidants (Vitamin C, peptides) | 7.8 | 9 | 8% | 0% | 92% |
| Hyaluronic acid serums | 7.8 | 9 | 8% | 8% | 83% |
| FP IV-VI; avoid harsh exfoliants or strong actives | 7.3 | 7.5 | 8% | 25% | 67% |
| Light moisturizers | 7.1 | 8 | 8% | 33% | 58% |

**Supplementary Table 3.** Appropriateness rankings for the presented algorithm.

|  |  |  | **PERCENTAGE** | | |
| --- | --- | --- | --- | --- | --- |
|  | **MEAN** | **MEDIAN** | **1-3** | **4-6** | **7-9** |
| How appropriate is the final structure of this treatment algorithm for addressing the key skin quality concerns (Skin Firmness, Surface Evenness, Skin Tone Evenness, Skin Glow)? | 8.4 | 9 | 0% | 0% | 100% |
| How well does the final algorithm incorporate the feedback provided during the discussion rounds? | 8.1 | 8 | 0% | 8% | 92% |
| How practical is this final algorithm for implementation in a typical clinical setting? | 8.3 | 9 | 0% | 17% | 83% |
| How well does the final algorithm address patient-centered outcomes such as satisfaction, aesthetic goals, and comfort with the treatments? | 6.9 | 8 | 17% | 0% | 83% |
| How well does the final algorithm address the key concerns of your region? | 8.2 | 8.5 | 0% | 17% | 83% |
| How well does the final algorithm incorporate the latest evidence and innovative treatments in dermatology? | 7.2 | 8 | 8% | 17% | 75% |
| How confident are you that this final algorithm will lead to improved patient outcomes in terms of skin quality? | 8.5 | 9 | 0% | 8% | 92% |
|  |  |  |  |  |  |
